# The effect of exogenous corticosterone on West Nile virus infection in Northern Cardinals (*Cardinalis cardinalis*)

**DOI:** 10.1186/1297-9716-43-34

**Published:** 2012-04-21

**Authors:** Jennifer C Owen, Ayaka Nakamura, Courtney AC Coon, Lynn B Martin

**Affiliations:** 1 13 Natural Resources, Michigan State University, Department of Fisheries and Wildlife, East Lansing, MI, USA; 2 Michigan State University, Department of Large Animal Clinical Sciences, East Lansing, MI, USA; 3 Michigan State University, College of Veterinary Medicine, East Lansing, MI, USA; 4 University of South Florida, Department of Integrative Biology, Tampa, FL, USA

## Abstract

The relationship between stress and disease is thought to be unambiguous: chronic stress induces immunosuppression, which likely increases the risk of infection. However, this link has not been firmly established in wild animals, particularly whether stress hormones affect host responses to zoonotic pathogens, which can be transmitted to domesticated animal, wildlife and human populations. Due to the dynamic effects of stress hormones on immune functions, stress hormones may make hosts better or poorer amplifying hosts for a pathogen contingent on context and the host species evaluated. Using an important zoonotic pathogen, West Nile virus (WNV) and a competent host, the Northern Cardinal (*Cardinalis cardinalis*), we tested the effects of exogenous corticosterone on response to WNV infection. Corticosterone was administered at levels that individuals enduring chronic stressors (i.e., long-term inclement weather, food shortage, anthropogenic pollution) might experience in the wild. Corticosterone greatly impacted mortality: half of the corticosterone-implanted cardinals died between five - 11 days post-inoculation whereas only one of nine empty-implanted (control) birds died. No differences were found in viral titer between corticosterone- and empty-implanted birds. However, cardinals that survived infections had significantly higher average body temperatures during peak infection than individuals that died. In sum, this study indicates that elevated corticosterone could affect the survival of WNV-infected wild birds, suggesting that populations may be disproportionately at-risk to disease in stressful environments.

## Introduction

Host exposure to stressors can have ramifications for the spread, persistence or emergence of pathogens [1]. In this paper, we consider a stressor to be any external factor that elicits a stress response that is characterized by impairment of an organism's ability to function normally, including their capacity to mount an immune response [2]. When an organism is exposed to a stressor, its resistance to infection may be reduced through this suppression of the immune system [3-5]. Lowered resistance may increase the chance of infection upon exposure to a parasite/pathogen and/or lead to greater amplification of the pathogen in the host. These effects might lead to higher morbidity of the infected animal, enabling vectors to obtain blood meals more effectively and become infected themselves, thus spreading the infection more easily. Whereas stress is well-associated with increased susceptibility to disease [6], links among stressors, their physiological mediators, and resistance to pathogens are not well established, especially in free-living animals. Stressors might take many forms in natural systems including inclement weather, food shortages, territorial conflicts, or threat of predation [2]. In human-modified landscapes, stressors may be even more common including such factors as noise, air, and water pollution, greater abundance of predators, and low-quality habitat and food availability [7-9].

In the US, a particularly important zoonotic pathogen is West Nile virus, or WNV. WNV causes morbidity and mortality in birds, mammals, domestic livestock and humans [10]. WNV has worldwide distribution; it has been found in North America, Australia, Europe, Asia and Africa [11]. For North American birds, WNV can cause substantial morbidity and mortality, particularly for some species of passerines [12-14], owls [15] and sage grouse (*Centrocercus urophasianus*) [16]. The enzootic cycle of WNV occurs between the vector mosquitoes, primarily *Culex *spp., and Passeriform birds [17]. Worldwide, WNV transmission primarily occurs in urban and agricultural environments [11,18-20]. The occurrence in urban environments is a pattern that has been mainly attributed to the abundance of *Cx. pipiens*, a mosquito that has adapted to anthropogenic habitats [20]. The potential role of the avian host in the clustering of WNV cases in human dominated landscapes has also received much attention [21-24]. Urban environments may support more competent host species than rural areas; competence being defined as the host's capacity to replicate and transmit a parasite. Indeed, species most competent for WNV (based on experimental infections) tend to be those that are common in urban environments [25]. The above observations may be biased because most experimental infection studies have been conducted on resident, peridomestic species [12,15,26-28], but see [29]. Moreover, in one of these studies [12], very small samples sizes (e.g. *n *< 5) were used for most species, making it difficult to draw significant conclusions about reservoir competence of some species relative to others.

The response of any vertebrate to stressors is mediated in large part by the hypothalamic-pituitary-adrenal (HPA) axis and the secretion of glucocorticoids (GCs) [2,30]. Although acute activation of the HPA axis can be protective against infections [4], chronic elevations of GCs tend to be detrimental, suppressing most, if not all, immune defenses [3,31,32]. In birds, the primary glucocorticoid is corticosterone, and in response to chronic stressors, plasma levels of corticosterone can be elevated many-fold [33].

Our goal in the present study was to directly test the effects of corticosterone elevation, to simulate exposure to a chronic stressor, on the response of a passerine to WNV infection. We predicted that administering exogenous corticosterone would elevate viremia (i.e., magnitude, or duration of virus circulation) due to immune suppression (an effect previously seen in chickens [34]), increase mortality and dampen the febrile response upon infection. We tested our hypothesis using the Northern Cardinal (*Cardinalis cardinalis*), a common North American passerine distributed across the entire eastern US, southern Canada and through northern Central America [35]. We chose this species for several reasons. First, cardinals are common visitors to bird feeders, thus come into close contact with humans. Second, cardinals are commonly infected with WNV and exhibit some of the highest natural seroprevalence rates measured [36-39]. Finally, cardinals account for a high proportion of the blood meals of the primary mosquito vectors in some urban areas [40], suggesting that they are a preferred host.

## Materials and methods

Adult Northern Cardinals (*n *= 35; 15 females and 20 males) were captured in central Michigan, United States, at several locations: Rose Lake State Wildlife Area in Bath, MI, and sites in East Lansing, Okemos, Williamston and Mason Townships in June and July 2009. Birds were target netted using mist nets and playback of male cardinal songs. Birds were transported to Michigan State University animal research containment facility and housed in individual cages (18 × 18 × 24in) within a biosafety level 3 animal room.

A blood sample (0.15 mL) was collected from each bird and was screened for West Nile virus antibodies (see below). Only birds confirmed as WNV-seronegative were held for the experiment. Two of these birds died prior to the experiment and one did not acclimate to captivity and was subsequently released. Cardinals (*n *= 19; 9 females and 10 males), stratified by sex, were randomly assigned to treatment (*n *= 10; 5 F and 5 M) and control (*n *= 9; 4 F and 5 M) groups. Birds were maintained under natural photoperiod specific to Michigan and fed ad libitum a mixed diet of sunflower and safflower seed mix, soft food mixture (puppy chow, carrots, and hardboiled eggs), mealworms and softened raisins.

On 25 July, all cardinals were inoculated subcutaneously with 10 000 plaque-forming units (pfu) of WNV strain NY385-99, using methods described by Owen et al. [29]. Blood was collected 10 h post-inoculation, and then 1 - 5 days post inoculation (dpi) at the same time of day as inoculation. Whole blood (0.05 mL) was diluted with BA-1 (0.45 mL; composed of Hank's M-199 salts, 1% bovine serum albumin, 350 mg/L sodium bicarbonate, 100 units/mL penicillin, 100 mg/L streptomycin, 1 mg/L Fungizone in 0.05 M Tris, pH 7.6), immediately placed on dry ice, and stored at -80°C.

All methods using animals in this study were approved by the Michigan State University animal use and care committee, IACUC protocol #01/10-007-00.

### Body mass and temperature

At each blood sampling, birds were weighed to the nearest 0.01 g and their body temperature to the nearest 0.1°C was taken using a BAT-12 microprobe thermometer and RET3 probe (Physitemp Instruments, Inc.) with tip diameter of .065", which was inserted into the cloaca. Birds were sampled in the same order every day to minimize the effect of time from initial disturbance on body temperature. Birds were also examined daily for any clinical signs of disease [41]. On 14 dpi, birds were bled (0.40 mL) and then euthanatized by CO_2 _inhalation.

### West Nile virus titers

WNV titers were determined in duplicate using Vero cell plaque assay in 6-well plates with a double 1.0% agarose overlay [42]. Plaques were counted on the 2^nd ^and 3^rd ^day after the second overlay with neutral red stain. Virus titer was log transformed and reported as log pfu/mL of serum with the detection threshold for the assays being 1.7 log pfu/mL serum. For two individuals on 3 dpi, viral plaques were too numerous to count at a dilution that would be equivalent to 12 log pfu/mL. However, when the assay was repeated at higher dilutions, sample titers fell between 11-12 log pfu/mL (lower values were likely due to viral degradation during prior freeze/thaw cycle). Therefore, a conservative 12.1 log pfu/mL titer was reported for these individuals.

### Plasma corticosterone

Plasma levels of corticosterone were measured using a competitive enzyme immunoassay (EIA; Assay Designs Inc., Ann Arbor, MI, USA) as described by Owen [43] and validated for use in cardinals (Martin unpublished observations). All samples for an individual were assayed on the same 96-well plate, in duplicate and in a random sequence. Concentration of corticosterone is reported in ng/mL of plasma. On 17 July 2009, after three-weeks in captivity, blood samples were collected from all birds to measure baseline corticosterone levels. Blood samples (0.075 mL) were collected via brachial or jugular venipuncture within three minutes of entering the housing room. On 20 July 2009, the treatment group (*n *= 10) was given corticosterone (Sigma C2505) implants (18 mm in length and 1.47 interior diameter) and the control group (*n *= 9) received empty implants as described by Soma et al. [44]. The implants were inserted subcutaneously under the right wing and the incision site was sealed with a dermal adhesive. Baseline CORT levels were sampled on 24 July (4 days post-implant; dpm) and then again on 5 August. Due to the amount of blood one can collect during a 2 week period of time, the stressful nature of collecting baseline corticosterone (i.e. large number of people quickly enter a room, grab birds from cages and collect blood sample), and limited access for most personnel during the active infection period, we only sampled for baseline corticosterone -3 dpm, 4 dpm (-1 dpi) and 15 dpm (n). It is known that corticosterone from implants can peak immediately after implant and then drop to non-detectable levels, even though the effect is pronounced for a period of time following implant [45]. The interassay coefficient of variation for the two plates was 0.04.

### Serology

Previous exposure to West Nile virus was assessed using a blocking ELISA (enzyme-linked immunosorbent assay) for specific WNV reactive antibodies as described by Blitvich et al. [46]. For a serum sample to be considered positive for WNV antibodies, it had to exhibit greater than 30% inhibition relative to the negative control, uninfected chicken serum (Vector Laboratories, Burlingame, CA, USA). Following the experimental infection, blood was collected at 14 dpi and assayed for WNV-specific antibody titer using plaque-reduction neutralization test (PRNT) [47]. The only modification to this method was the use of 6-well plates and inoculating those plates with 0.1 mL of the virus-diluted blood sample. Antibody titers are reported as the serum dilution that inhibited 90% of the virus plaque formation.

### Data analysis

Data were analyzed using Chi-square tests, independent sample *t*-tests, or repeated measures ANOVA with treatment group as independent variables and physiological measures as response variables. When the sphericity assumption was violated in the ANOVA tests, the Greenhouse-Geisser test was used. All data presented as described above passed assumptions of normality. Paired t-tests were used for all post-hoc comparisons and alpha adjusted for multiple comparisons. Pearson correlation analyses were used to detect relationships between corticosterone concentration, body temperature, and virus titer at the various sampling points. Baseline plasma corticosterone values were reported and analyzed as the difference in concentration from pre-implant levels to control for individual differences in starting concentration. We could not test whether capture site impacted host responses due to low statistical power. An alpha level of 0.05 was set for all analyses and in each case; the derived *P*-value refers to two-tailed tests. Analyses were performed with SPSS 18.0 (SPSS, Inc. Chicago, IL, USA).

## Results

### Viremia

**Figure 1:**
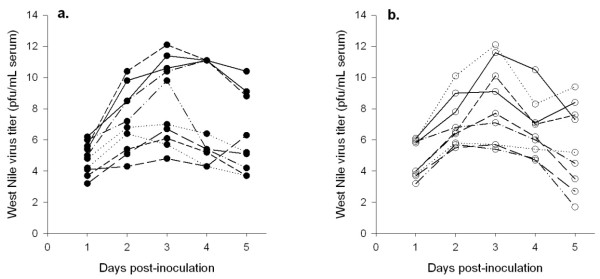
**Viremia profiles for (a) corticosterone-implanted and (b) empty-implanted Northern Cardinals infected with 10 000 pfu/mL of West Nile virus**.

All birds exhibited detectable levels of WNV post-infection (Figure 1). Peak viremia occurred on 3 dpi (Table 1) with individual titers ranging from 4.8 to more than 12.1 log pfu/mL of blood. Virus titers during the 5-day period post-inoculation did not differ between empty and corticosterone-implanted cardinals (F_1, 15 _= 0.07, *p *= 0.80) or between sexes (F_1, 15 _= 0.12, *p *= 0.74). Peak titer also did not differ between treatments (*t *= 0.42, *df *= 17, *p *= 0.68). However, peak titer did differ between birds that survived and those that died during the experiment (*t *= 3.38, *df *= 17, *p *= 0.004; Table 1): birds that died circulated significantly more virus. To compare total viremia, integrals of titers at each time interval were calculated for each individual. However, there was no difference in total WNV titers between the two groups (Mann Whitney U = 33.0, *p *= 0.54). Infective duration could not be assessed because all birds were still viremic on the last day of sampling.

**Table 1:** Daily mean (± SD) viremia titers (log pfu/mL serum) for Northern Cardinals (*Cardinalis cardinalis*) implanted with either corticosterone (*n *= 10) or an empty silastic tubule (*n *= 9) and inoculated with a 10 000 pfu of West Nile virus.

	Min (above 1.69 log pfu*), Max				Mean (SD)		
	**Corticosterone**		**Placebo**		**Corticosterone**		**Placebo**

	**Survived** ***n *= 5**	**Died** ***n *= 5**	**Survived** ***n *= 8**	**Died** ***n *= 1**	**Survived** ***n *= 5**	**Died** ***n *= 5**	**Survived** ***n *= 8**

1 dpi	3.2, 6.0	4.8, 6.2	3.2, 6.1	6.0	4.24 (1.06)	5.4 (0.55)	4.53 (1.16)

2 dpi	4.3, 7.2	6.8, 10.4	5.5, 9.0	10.1	5.68 (1.13)	8.8 (1.39)	6.58 (1.19)

3 dpi	4.8, 9.8	7.0, 12.1	5.4, 11.6	12.1	6.62 (1.9)	10.3 (1.96)	7.67 (2.28)

4 dpi	4.3, 5.4	6.4, 11.1	4.7, 10.5	8.3	4.9 (0.6)	10.2 (2.1)	6.29 (1.86)

5 dpi	3.7, 6.3	5.2, 10.4	1.7, 8.4	9.4	4.6 (1.11)	8.74 (2.23)	5.06 (2.46)

Minimum and maximum viral titers are reported for all birds with detectable levels on the given day. dpi = days post-injection. *1.69 log pfu is detection limit of assay.

### Mortality

**Figure 2:**
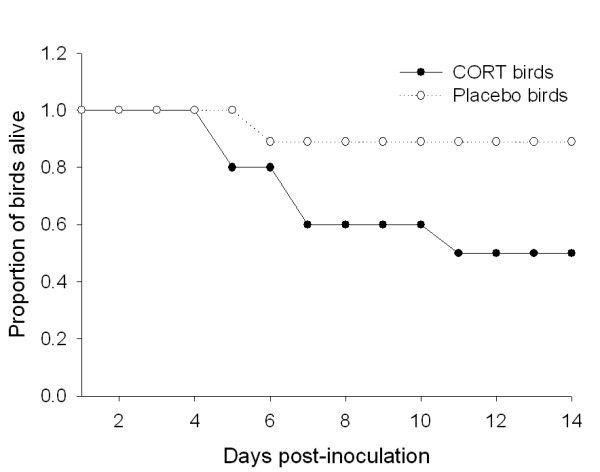
Effects of corticosterone or empty-implants on survival of Northern Cardinals infected with 10,000 pfu/mL of West Nile virus

Five of the 10 corticosterone-implanted cardinals, but only one of the 9 empty-implanted birds, died during the experiment (Figure 2). Previous experimental infection studies with cardinals conducted under identical protocols [27] resulted in a 22% (2 of 9 individuals) mortality rate. Given this expected mortality rate for the empty-implanted birds, the mortality rate observed in the current experiment (11%, 1 in 9 individuals) did not differ significantly (*X^2 ^*(1, N = 18) = 0.40, *p *= 0.53). However, a mortality rate of 50% (5 of 10 individuals) for the corticosterone-implanted birds trended toward higher than the empty-implanted birds in current experiment (*X^2 ^*(1, N = 19) = 3.32, *p = *0.069).

### Body temperature

**Figure 3:**
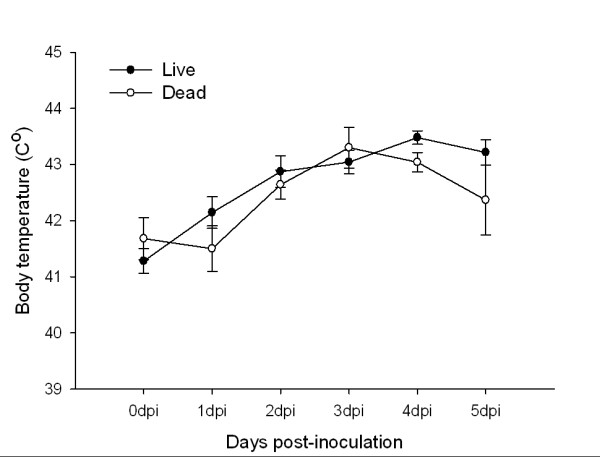
**Body temperature (°C; mean ± 1SE) of Northern Cardinals that survived or died from West Nile virus infection for the five days post-inoculation**.

Regardless of treatment or survival, body temperature increased between 0 dpi and peak viremia (2-5 dpi; 1 dpi was excluded because it was measured at a different time of day than all others; *t *= -7.68, *df *= 17, *p *< 0.001). However, birds that survived the infection exhibited higher temperatures than individuals that died (Figure 3; *F*_4, 68 _= 2.89, *p *= 0.03). A second analysis further supported this result: taking the average of body temperature during the early viremic period (2-5 dpi) and subtracting this mean from body temperature on 0 dpi, surviving birds exhibited a larger febrile response during viremic period than birds that died (*t *= 2.36, *df *= 17, *p *= 0.03). There was no difference between corticosterone and control groups (*t *= 0.27, *df *= 17, *p *= 0.79), nor was there an effect of corticosterone on body temperature over the entire viremic period (*F*_1, 17 _= 2.26, *p *= 0.15).

### Morbidity

**Figure 4:**
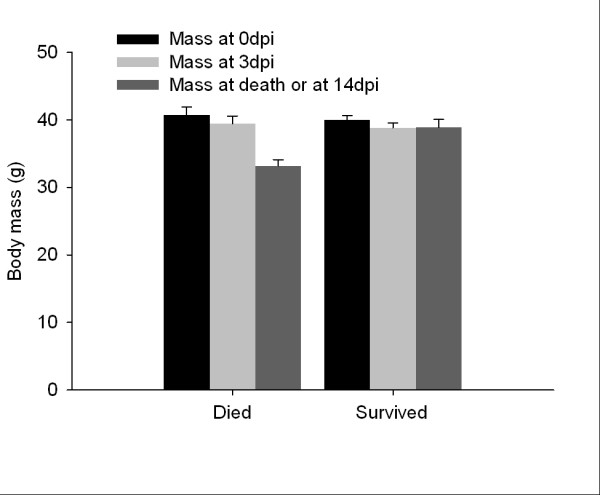
**Mean body mass (g; mean ± 1SE) of Northern Cardinals that either died or survived a West Nile virus infection at 0 days post-inoculation (dpi), peak viremia at 3 dpi, and either at death, or 14 dpi**.

Birds that died lost an average of 18% of their original (0 dpi) body mass compared to 2% mass loss in individuals that survived (Figure 4; F_1, 17 _= 34.0, *p *< 0.001), but corticosterone implants did not affect mass loss (F_1, 17 _= 0.16, *p *= 0.70). Qualitative differences in clinical signs of disease between birds that survived and died were observed including weakness, lethargy, anorexia, and ruffled feathers. Some affected birds were unable to perch and laid in sternal recumbency on the cage floor. Watery stool was also observed on cage floors. A few of the birds demonstrated tremors along with other clinical signs. Upon necropsy, the birds that died from WNV had protruding keels with very little furcular fat. Interestingly, one control bird exhibited severe neurological disorder and had difficulty balancing on the perch, but ultimately recovered and survived to the end of the experiment.

### Corticosterone

**Figure 5:**
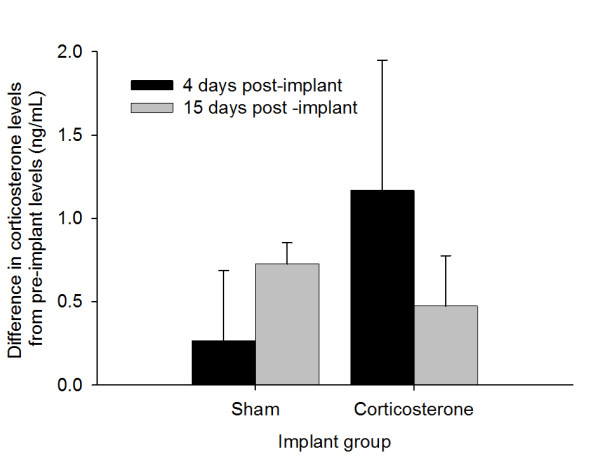
**Changes in plasma corticosterone levels in empty-implanted and corticosterone-implanted Northern Cardinals from pre-implant levels to 4 and 15 days post-implant**.

Plasma levels of corticosterone did not differ between groups at pre-implant sampling (*t *= 0.86, *df *= 17, *p *= 0.40). Plasma corticosterone levels increased by 4 dpm in both groups but the increase was greater in the corticosterone-implanted group, though not statistically significant (Figure 5; *t *= -0.98, *df *= 17, *p *= 0.34). Also, corticosterone levels for birds that died during the experiment did not differ from those that survived, either prior to (*t *= -1.63, *df *= 16, *p *= 0.12) or post-implant (*t *= -0.63, *df *= 17, *p *= 0.54). Virus titer on 1 dpi and 5 dpi were negatively correlated to plasma corticosterone concentration on -1 dpm (1 dpi, *r *= -0.50, *n *= 18, *p *= 0.036; 5 dpi *r *= -0.54, *n *= 17, *p *= 0.025). There was no relationship between corticosterone on -1 dpm and virus titer on 2 - 4 dpi.

## Discussion

Cardinals with exogenous corticosterone had 4.5 times greater mortality than empty-implanted birds. While this finding was not statistically significant (*p *= 0.069) we would argue this difference is biologically significant. However, corticosterone implants did not affect viral titers, body temperature, body mass or circulating corticosterone levels. These effects are consistent with results from other infection studies with other pathogens and hosts [48]. For example, brown trout (*Salmo trutto L*.) naturally infected with several different bacterial or fungal pathogens had a dose-dependent increase in mortality when administered cortisol through intra-peritoneal injection [49]. However, mice given exogenous corticosterone in their drinking water and then experimentally inoculated with a highly pathogenic strain of avian influenza (A/Vietnam/1203/04) did not exhibit differences in mortality compared to non-treated controls [50], though there were corticosterone-effects on mass with untreated mice losing more mass than corticosterone administered individuals. Likewise, chickens exposed to corticosterone through their drinking water did not exhibit heightened mortality to WNV, which may be attributed to their overall low viremia regardless of treatment (peak viremia 3-5.0 pfu/mL of blood; [34]). The lack of effects of corticosterone on viremia in our cardinals is also contrary to some other studies [34,51,52]. Chickens infected with WNV and treated with corticosterone and with corticosterone and the toxicant, resmethrin, exhibited heightened, and prolonged viremia compared to controls and resmethrin-only treated chickens. Likewise, domestic dogs infected with WNV and administered a corticosteroid analog, methylprednisolone acetate, exhibited a more intense (on average 40× greater) and longer viremia than the untreated group [52].

Collectively, these results suggest corticosterone effects on viremia and virus-induced mortality are diverse; however, these studies may not be directly comparable because of the use of different pathogens, hosts, dosages, and routes of administering corticosterone as well as the use of synthetic GCs, which have different receptor binding chemistry. In our study, corticosterone levels may have been too low to affect viremia but sufficient to impact survival in some individuals. Perhaps had we assessed viremia duration, corticosterone effects would have been detectable. On the other hand, a previous study in gray catbirds (*Dumetella carolinensis*) found that peak WNV titer is positively correlated to viremia duration (*r *= 0.40, *n *= 52, *p *= 0.003; Owen unpublished data).

An intriguing additional possibility is that species differ systematically in how corticosterone affects viremia. The mortality without high viremia outcome in our study is somewhat surprising because viremia is often positively associated with mortality both among and within species; typically, WNV-infected birds do not survive titers that exceed 9.0 log pfu [12,15,28,53]. Furthermore, in our study, all birds that died had peak titers of 10 log pfu or greater, similar to our previous experimental infection study [27]. However, contrary to other studies, some individuals in our study had peak titers greater than 9.0 log pfu/mL and survived the experiment (Table 1). These high titers in surviving cardinals is particularly interesting with regards to their ability to serve as a reservoir for WNV. Based on reservoir competence index estimates [12], cardinals from this study (assuming a minimum index of 1.0, a conservative estimate because we did not assess viremic duration) resemble species considered highly competent for WNV, such as American robins (*Turdus migratorius*) and corvids [25]. Although reservoir competence does not account for vector feeding preference, in which robins are a considerably more preferred host than cardinals, our data suggest that populations and maybe even individuals, differ in their competency to replicate and transmit viruses to vectors. In other words, some species, including cardinals, might be particularly important amplifying hosts for WNV, especially in environments, such as urban areas, with large amounts of endocrinological or immunological variation.

Another unexpected outcome of the current study was a lack of measurable elevation of circulating corticosterone post-implantation. Unlike some other corticosterone implant studies, we did not leave open the implant ends, which may have impeded diffusion of the hormone. However, based on visual inspection of the implants throughout the experimental period, corticosterone was being released: at 14 days post-implant, each implant was one half to two-thirds full with corticosterone. Moreover, other studies too have found no change in circulating corticosterone post-implant but with strong effects on other physiological systems [45,54]. Finally, in a pilot experiment testing the effect of having implants with both ends sealed versus one end sealed in the baseline corticosterone levels in American robins (*Turdus migratorius*) it was found that corticosterone was 100× and 200× greater 1 day post-implant, respectively (Owen unpublished observations). Hence, it doesn't appear that having both ends sealed prevented the release of corticosterone and, furthermore, it was likely a more biologically relevant increase in corticosterone. Most importantly, there was a significant negative impact of corticosterone on survival in the present study; no other factor differed between treatment groups.

Corticosterone implants also did not affect body temperature or antibody responses to WNV. However, during the viremia period (2 ~ 4 dpi), birds that died had lower body temperatures than birds that survived. These increases in body temperature of 1-2°C are consistent with febrile responses found in other birds [55,56]. The importance of the febrile response in reducing morbidity and mortality of infected animals has received some support see review [57], but this is one of the first experimental examples in a wild bird that fever is related to survival of viral infection. Suppressive effects of corticosterone on the release of pyrogenic cytokines and production of prostaglandins, which mediate elevations in body temperature, are well-known [58,59], so it was surprising to have observed no effects of the implants on body temperature. However, capture, and handling of birds prior to temperature measurements may have obscured corticosterone effects on fever. Future experimental infection studies could monitor body temperature remotely (via telemetry) [60].

In summary, corticosterone implantations increased Northern Cardinal mortality to WNV by 450%, but did not affect viremia, mass or body temperature. However, further research is necessary to determine whether these results can be extrapolated to free-living individuals and whether subtler changes in corticosterone may have different effects on mortality and viremia. Presently, our data suggest populations in stressful environments may suffer disproportionate mortality if exposed to WNV. However, competence for WNV would seem to be invariant or even reduced in such environments, as birds with very high viremia would be expected to die quickly, although in the current study most of the birds survived through the first 5 days of infectious when a bird is most viremic. We emphasize caution in making such a strong conclusion though, as our data may be unrepresentative of cardinal responses to WNV in the wild. Free-living populations vary in their access to quality food resources, and those with greater access may be able to endure stressors and perhaps serve as more competent hosts for WNV in spite of elevated corticosterone. Indeed, field work to assess whether and how natural and anthropogenic stressors affect the role of cardinals and other species and populations in the dynamics of WNV and other parasites is a critical yet understudied area in disease ecology.

## Abbreviations

Dpm: Days post implant; dpi: Days post infection; WNV: West Nile virus; pfu: Plaque-forming units.

## Competing interests

The authors declare that they have no competing interests.

## Authors' contributions

JO conceived (with LM) of the study, designed the experiment, directed the experimental infection studies, conducted statistical analyses and drafted the manuscript. AN carried out the field study, experimental infection study and conducted the laboratory work. CC participated in the laboratory work and writing of the manuscript, LM conceived (with JO) of the study, participated in its design, and coordination and helped to draft the manuscript. All authors read and approved the final manuscript.

## Author's information

JO is an Assistant Professor in the Department of Fisheries and Wildlife in College of Agricultural and Natural Resources and in the Department of Large Animal Clinical Sciences in College of Veterinary Medicine at Michigan State University. JO specializes in the experimental infection studies with wild birds. AN is a 4^th ^year veterinary student in the College of Veterinary Medicine at Michigan State University and conducted this research during a summer endowed -research program, CC is a doctoral student at University of South Florida under the direction of LM, and LM is an Assistant Professor at University of South Florida and specializes in how stress mediates immune function in wild birds.
